# Utilization of Fermented Rice Milk as a Novel Coagulant for Development of Paneer (Soft Cheese)

**DOI:** 10.3390/foods8080339

**Published:** 2019-08-12

**Authors:** Rasool Khan Amini, Md Zohurul Islam, Yutaka Kitamura, Mito Kokawa

**Affiliations:** 1 Graduate School of Life and Environmental Sciences, University of Tsukuba, 1-1-1, Tennodai, Tsukuba, Ibaraki 305-8572, Japan; 2 Faculty of Life and Environmental Sciences, University of Tsukuba, 1-1-1, Tennodai, Tsukuba, Ibaraki 305-8572, Japan

**Keywords:** rice milk, saccharification, fermentation, micro wet milling, paneer

## Abstract

In this study, fermented rice milk was used as a novel coagulant for a type of soft cheese named as paneer. Rice milk was produced by a wet milling system in a process where brown rice was first soaked in water at a ratio of 1:2 (*w*/*w*), then milled by micro wet milling. Rice milk was pasteurized and gelatinized followed by the saccharification and lactic acid fermentation process. Paneer was produced using whole dairy milk mixed with 10%, 20%, and 30% of simultaneous saccharified and fermented (SSF) rice milk as a coagulant, and was analyzed for its physicochemical, microbial, and sensory properties. The results indicated that fermented rice milk has constructive effects on the physicochemical properties, texture, and shelf life of paneer, as there were no obvious defects observed for up to 12 days of storage at 4 °C. The sensory evaluation revealed that the acceptability score of the samples containing rice milk reduced slightly compared to the control samples. No significant differences (*p* ≤ 0.05) were observed among all the paneer samples incorporated with different percentages of rice milk, and the product was rated acceptable.

## 1. Introduction

Paneer is a soft variant of cheese obtained by heat and acid coagulant of dairy milk and has a spongy texture, mildly acidic flavor, and milky white color. Paneer is widely consumed in South Asian countries such as Afghanistan, Bangladesh, India, and Pakistan. It is usually served for breakfast or used in culinary dishes [1]. Paneer contains low amounts of lactose (approximately 2%), and has a high percentage of fat (25%), ash (1.8%), and protein (20%) content, especially when it is prepared from buffalo milk [2]. Paneer has a high moisture content (52%), providing optimal conditions for bacterial growth, leading to a shorter shelf-life [3]. The shelf-life of paneer is only one day at room temperature and 7 days in refrigerator temperature [4]. Many attempts have been made to enhance the shelf life of paneer, such as using different packaging material [4], use of different spices [5], addition of plant essential oil [3], and applying hurdle technology [4], etc. Production and development of different types of paneer such as soy paneer, paneer from peanut milk, filled paneer, vegetable-impregnated paneer, and ultrafiltration have also been previously attempted [6,7,8,9].

In paneer processing, several acids are used for coagulation, such as citric acid, tartaric acid, lactic acid (LA), malic acid, and acetic acid, with heat to break down the casein structure as curd form which is further collected with a muslin cloth and served as fresh paneer product [10]. Conventionally, paneer is produced using sour fruit juice, such as lemon amla, as a coagulant [10]. The amount and type of acid plays an important role in paneer quality [8], and using alternative coagulants such as fermented whey and buttermilk may be effective in producing paneer with a better texture and improved health benefits [11].

In this study, we produced paneer from dairy milk mixed with fermented brown rice milk (micro-milled rice slurry) as a coagulant to increase the nutritional value of paneer, and also to produce an acceptable product to utilize rice milk. Studies have shown that brown rice is more nutritious as it contains additional nutrients lacking in white rice and is low in calories compared to white rice [12,13] and therefore we micro wet milled (MWM) brown rice to produce rice milk. This system produces rice milk from raw brown rice without prior polishing or cooking. Advantages in this system are that it produces less heat during operation, preserving heat-sensitive nutrients. The MWM system can mill rice into particle sizes less than 2 µm, which can then be used as a rice milk beverage [14]. The MWM rice milk was subject to LA fermentation to produce a novel coagulant for paneer processing. LA fermentation of rice milk can improve the nutritional value by synthesizing the mineral, vitamins, and amino acid [15]. In addition, it is expected to improve the paneer texture and reduce the amount of lactose as the demand for lactose-free food is growing globally, due to its health implication for lactose-intolerant people [16].

Conventionally, the fermentation of starchy material such as rice is done after a long process of gelatinization, liquefaction and enzymatic saccharification to reducing sugars. Contrarily, saccharification of starch and fermentation can be done simultaneously to avoid energy and time consumption. Studies have shown that simultaneous saccharification and fermentation (SSF) is a simple method for fermentation of starchy material to LA and ethanol [17].

The objectives of this study are to incorporate rice milk in paneer making, and also to analyze the appropriate method of saccharification and fermentation for rice milk fermentation, as no study has mentioned the use of fermented rice milk in paneer processing.

## 2. Materials and Methods

### 2.1. Sample Collection

Brown rice (Koshihikari) was collected from Ibaraki, Japan, and used for producing rice milk (rice slurry). Dairy whole milk was procured from Takanashi, Iwate, Japan.

### 2.2. Production of Rice Milk

**Figure 1:**
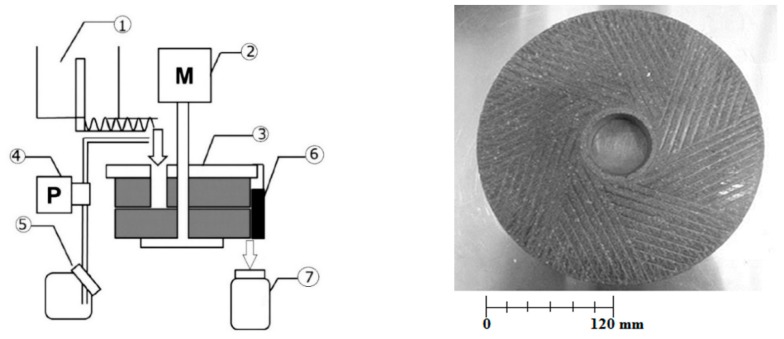
Schematic diagram of the micro wet milling system. (1) Rotary rice feeding equipment, (2) Motor, (3) Milling stone, (4) Tubing pump, (5) Water tank, (6) Rubber Spatula, (7) Receiver. Picture of the lower stone of mill [14].

Rice milk (MWM rice slurry) was prepared from brown rice. Brown rice was soaked in water at a rice to water ratio of 1:2 and kept at 2 °C for 5 h. After soaking, rice was coarsely ground with a mixer (Hamilton Beach-number, Japan) for 3 min and then milled with MWM to produce rice milk according to the method described by Koyama and Kitamura [14] with slight modifications. The MWM process flow diagram is shown in Figure 1. The rice coarse slurry mixture was kept in a rotary feeding machine and fed to the milling stone at a rate of 18 g min^−1^. Concurrently, the tubing pump fed water at a rate of 40 mL min^−1^ to the stone mill. The rotational speed of the milling stone was 50 rpm. The stone mill has grooves, an exterior surface area of 207 cm^2^, and a radius of 12 cm. The larger contact surface of the mill produces smaller particle sizes and the optimum milling conditions were considered to obtain the smallest particle size. Rice milk was collected simultaneously in the collection tank as the course rice slurry was milled.

### 2.3. Preparation of Fermented Rice Milk

The raw rice milk was first gelatinized and liquified by 0.1% alpha-amylase enzyme at 90 °C for 30 min. After the liquification process, four different conditions were applied for saccharification and fermentation. The first samples were prepared with simultaneous saccharification and fermentation (SSF) processes, where 0.1% glucoamylase (Glucozyme) and commercial lactic acid bacteria (LAB) starter (0.0016 g) fermenting culture containing *Lactobacillus planetarium* spp. (Bactoferm^®^ Vegestart 60, CHR HANSEN Biosystems, Horsholm, Denmark) was added to 200 g of rice milk, and saccharification and fermentation were done simultaneously. The second set of samples, saccharification then fermentation (SF), were prepared with the conventional approach, where the rice milk was saccharified prior to fermentation. For SF, rice milk was saccharified with 0.1% glucoamylase and kept at 55 °C for 12 h before fermentation. The third type of samples, without saccharification (WS), were prepared from gelatinized and liquefied rice milk and were fermented directly without saccharification. Fourthly, samples were prepared by adding 0.5% of CaCO_3_ to SSF for evaluating the effect of pH on rice milk fermentation (CSSF). The same fermentation conditions were used for all samples. Fermentation was carried out in 500 mL Erlenmeyer flasks in a shaker incubator at a speed of 100 rpm for 72 h at 37 °C in triplicates. Samples were collected at 12 h, 24 h, 48 h, and 72 h, and their properties were analyzed.

### 2.4. Determination of Physical Properties of Rice Milk

The particle size of the rice milk was determined by a laser diffraction particle analyzer (SALD-2200, Shimadzu, Japan) in wet measurement mode. Data were expressed as average particle size (D50). The viscosity of the rice milk was measured using a Brookfield-type viscometer (DV-E, Brookfield Engineering) at 25 °C and at a speed of 12 rpm. Glucose amount was determined by a C_2_ test kit (Wako Pure Chemical Industries, Osaka, Japan).

### 2.5. Determination of Total Reducing Sugar, pH, and LA

Total reducing sugar was analyzed with a total reducing sugar kit (Megazyme, Wicklow, Ireland). Samples were diluted with a sample to distilled water ratio of 1:10 ratio and quantified at the absorbance of 340 mm with a spectrophotometer (JASCO V630 Japan). The pH of the samples was determined with a HORIBA scientific pH meter. LA content was measured by titration with 0.05 molL^−1^ NaOH, according to the method described by Maslanka et al. [18].

### 2.6. Microbial Analysis

Total LAB count and the total microbial count were measured for fermented rice milk and paneer samples using Petrifilm plates (3 M, St. Paul, MN, USA), and plated for aerobic counts using Petrifilm™ Aerobic. Samples were diluted up to 10^10^ with 0.9% saline solution, then 1 mL of selected dilutions were plated for aerobic counts using Petrifilm™ Aerobic Count plates and 3 M Petri films LAB. The plates were incubated at 37 °C for 48 h then counted for total LAB and total microbial count. The results are presented as log colony-forming unit (CFU) mL^−1^ of the sample, as instructed by the manufacturer [19].

### 2.7. Development of Paneer

Paneer was prepared with the method stated by Kumar et al. [1], with slight modifications. Dairy milk was heated to 82 °C for 5 min then cooled to 70 °C. Fermented rice milk was heated to 70 °C and added at the percentage of 10%, 20%, and 30% to dairy milk as a coagulant containing LA to lower the milk pH below the milk protein isoelectric point of 4.6 for successful coagulation [20]. Milk was continuously stirred until the whey and curd were separated. The coagulated milk was rested for 5 min and then drained using a muslin cloth. The coagulate was then pressed by 4 kg weights for 15 min. Paneer samples were sliced into 20 g cubic shapes and were packaged individually in sterilized zip-lock plastic bags and stored at 4 °C for 12 days. The control samples were prepared with 2% citric acid as a coagulant instead of rice milk.

### 2.8. Determination of Moisture Content, Total Yield, and Total Acidity of Paneer

The moisture content of the samples was determined by drying method the difference in weights before and after they were oven dried at 105 °C for 24 h, according to official methods [21].The yield of paneer is the difference of total dairy plus rice milk used and the whey obtained [8]. Total acidity was measured by titration, where 3 g of sample was diluted in 120 mL distilled water and homogenized with a mixer. Titration was carried out with an automatic potentiometric titrator using 0.05 mol L^−1^ NaOH.

### 2.9. Color Measurement of Paneer

The samples from fresh and stored paneer were subjected to color measurements. The picture of the samples were taken with a color scanner (Epson GT-X980) and analyzed using MATLAB software [22]. The color value was recorded as RGB (red, green, blue) from 0 to 255 values, and was converted to Commission Internationale de l’Elcairage (CIE) color parameter as L*, a*, and b* values using EasyRGB software (IRO group Ltd., Triest, Italy).

### 2.10. Texture Measurement

The texture of paneer was analyzed with a texture analyzer (EZ-SX with 100 N load cell, Shimadzu, Japan). Compression tests were carried out using a 20 mm cylinder probe with a speed of 1 mm s^−1^. Compression results were collected and analyzed using the software (Trapezium 1.4.2) attached to the texture analyzer obtaining the hardness of paneer samples. Hardness is the peak force of the first compression when the probe compressed the sample [23].

### 2.11. Storage Test

A storage test was carried out to determine the physiochemical and biological changes in paneer properties during storage. The paneer samples (0%) rice milk as control with 2% citric acid, 10%, 20%, and 30% *v/v* rice milk and dairy milk were prepared and stored at 4 °C for 12 days. The samples were evaluated for moisture, microbial, color, and texture properties on days 1, 3, 6, 9, and 12 of storage.

### 2.12. Sensory Evaluation

Sensory evaluation was determined using a nine-point hedonic scale [24]. Paneer samples were freshly prepared and stored at 4 °C until the test. The test was done on the same day when paneer samples were prepared. The samples temperature was equilibrated with room temperature before the test. The organoleptic properties were evaluated by 20 semi-trained panelists for appearance, taste, texture, smell, and overall acceptability.

### 2.13. Statistical Analysis

The experiment was replicated three times, and the data was collected and analyzed with statistical software JMP 2.0.1 (SAS Institute Japan Ltd., Tokyo, Japan). Tukey HSD test *α =* 0.05 was used to differentiate the differences between analysis means.

## 3. Results and Discussions

### 3.1. Physical Properties of Rice Milk

Rice milk was produced from brown rice using the MWM process. The optimum milling condition was determined by obtaining the minimum particle size of the produced rice milk. During milling of rice at a feeding rate of 18 g min^−1^, a feeding rate of water 40 mL min^−1^, and maximum rotational speed 50 rpm of the MWM gives the particle size (D50) of 6.25 µm. The results indicated that the minimum particle size of the rice milk show characteristics similar to dairy milk and can be used for fermentation and then used as a coagulant for the production of soft cheese. The viscosity of the rice milk was 13.5 ± 2 mPa s. The glucose concentration of the MWM rice milk was 8.2 g L^−1^. Our results were in accordance with the results reported by Koyama et al. [14], who measured the physical properties of rice slurry produced by MWM.

### 3.2. Properties of Saccharified and Fermented Rice Milk

#### 3.2.1. pH and LA Production

**Figure 2:**
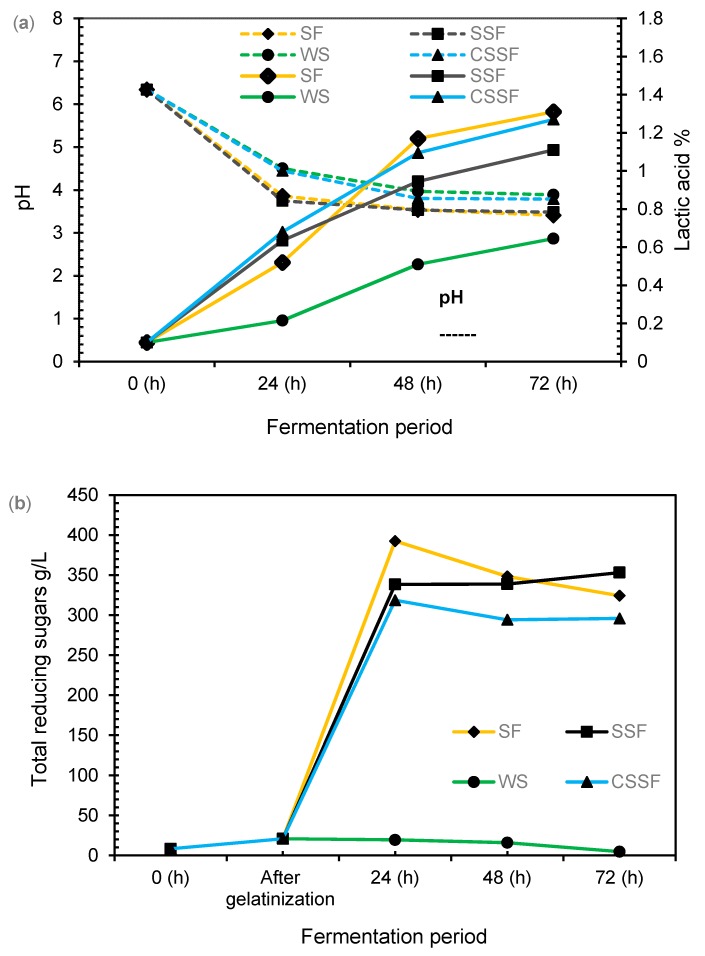
(**a**) Changes of pH during fermentation and (**b**) changes in reducing sugars during simultaneous saccharifications and fermentation (SSF) (∎), Saccharification then fermentation (SF), (◆) addition of CaCO_3_ (CSSF), (▲) and without saccharification (WS) (●).

The fermentation of rice milk was carried out with LAB for 72 h. Changes in pH values indicated the fermentation process. Figure 2a shows the fluctuation of pH and LA values during the fermentation period. The initial pH of rice milk was 6.3, which decreased rapidly in the first 24 h, to 3.8 in SF, 3.7 in SSF, 4.5 in WS, and 4.4 in CSSF due to the increase in LA from an initial 0.01% to 0.52% in SF, 0.63% in SSF, 0.21% in WS, and 0.68 in CSSF. The pH reduction slowly stabilized from 24 h to 48 h as a result of high acidity affecting LAB growth [25]. The pH gradually decreased to 3.4 in SF, 3.5 in SSF, 3.8 in WS, and 3.7 in CSSF after 72 h. Although LA production was slow, it slightly increased to 1.3% in SF, 1.11% in SSF, 0.65% in WS, and 1.27% in CSSF, respectively after 72 h. Samples with no saccharification WS showed higher pH and lower LA due to low reducing sugar as carbon source for LAB. The effects of CaCO_3_ on CSSF was not significant as it increased the pH at 24 h of fermentation, but then decreased at 48 h. The results were not significantly different with SF and SSF at the end of fermentation. Similar results were reported [26] for the simultaneous liquefaction, saccharification, and LA fermentation of barley starch.

#### 3.2.2. Changes in Reducing Sugars

In order to convert rice milks’ starch to simple sugars for bacterial consumption, enzymatic saccharification was performed to alter starch to reducing sugars. The changes in reducing sugars during the fermentation period are presented in Figure 2b. The initial amount of reducing sugars in raw rice milk was 8.2 g L^−1^, which increased to 20.8 g L^−1^ in all the samples after sterilization and gelatinization of rice milk at 90 °C for 30 min. SF samples were incubated at 55 °C with the addition of 0.1% glucoamylase for 12 h. The total reducing sugars in SF samples increased to 384.8 g L^−1^ after 12 h saccharification. SSF samples were subjected to saccharification and fermentation simultaneously at 37 °C, the amount of reducing sugars increased to 272.5 g L^−1^ after 12 h saccharification and fermentation. Reducing sugars in WS samples (20 g L^−1^) did not change during the first 12 h.

During fermentation, reducing sugars slightly increased to 392.5 g L^−1^ in SF, 338.3 g L^−1^ in SSF, and 318.5 g L^−1^ in CSSF, but slightly decreased to 19.3 g L^−1^ for WS after 24 h. As bacteria growth increased during fermentation, their consumption of sugar also increased, indicated by the reduction of reducing sugars. The reducing sugars amount decreased sharply to 324.2 g L^−1^ in SF, but in SSF samples, reducing sugars still increased to 353.1 g L^−1^ due to the presence of glucoamylase. The change of reducing sugars was not significant during 48 h and 72 h in CSSF samples; as it slightly increased from 294.1 g L^−1^ to 295.7 g L^−1^. Samples with no saccharification process (WS), reducing sugars reduced sharply to 4.6 g L^−1^ after 72 h of fermentation. Results suggested that there was no significant difference in the production of reducing sugars by the two methods SF and SSF. Therefore, samples from the SSF method showed high efficiency due to less energy and time consumed. The results were comparable with the study carried out on saccharified rice to produce a yogurt-type product [27].

#### 3.2.3. Total Bacterial Count as LAB

**Figure 3:**
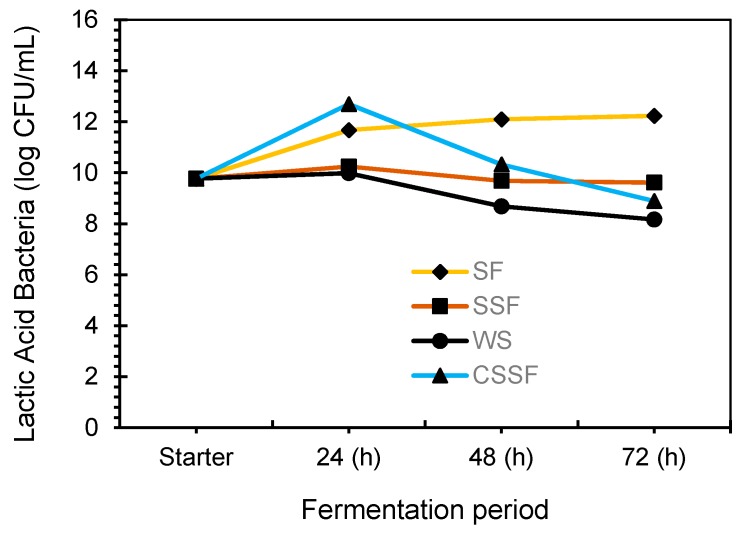
Growth curve of lactic acid bacteria during simultaneous saccharification and fermentation (SSF) (∎), saccharification then fermentation (SF) (◆), addition of CaCO_3_ (CSSF) (▲), and without of saccharification (WS) (●).

Fermentation of rice milk was carried out with LAB starter. The production of LA and bacteria growth was observed at 24 h of fermentation by the lowering of the rice milk pH. The bacterial growth curve was higher and steady at 24 h of fermentation in SF samples (11.67 log CFU mL^−1^) due to the abundance of reducing sugar by 12 h pre-saccharification process. The growth curve was maximized up to 72 h at the end of the fermentation process, as presented in Figure 3.

Total bacterial count in SSF was stable up to 24 h (10.24 log CFU mL^−1^), 48 h (9.68 log CFU mL^−1^), and slightly decreased to 9.61 log CFU mL^−1^ after 72 h of fermentation. After 24 h of fermentation, the pH of rice milk dropped to 3.75 from an initial pH 6.34. Consequently, a pH lower than 5 suppresses the growth of LAB [28]. Therefore, additional samples (CSSF) were prepared, with 0.5% of CaCO_3_ added to SSF samples to study the effect of pH alteration on bacterial growth during the fermentation process. The pH increased from 3.75 to 4.45 by the addition of 0.5% CaCO_3_ and had a positive effect on the bacterial growth, which increased to 12.69 log CFU mL^−1^ at 24 h fermentation. However, after 48 h of fermentation, the pH observed reduced to 3.80 and bacterial count also decreased to 10.32 log CFU mL^−1^. The bacterial growth of CSSF samples decreased rapidly compared to SF and SSF at 72 h of fermentation. The effect of 0.5% of CaCO_3_ was highly effective at 24 h of fermentation but there was no significant difference in pH of all the samples after 72 h. In addition, the final amount of LA, pH, and reducing sugar between SF and SSF were not significantly different. Nevertheless, the SSF method consumes less energy and time compared to SF, CSSF, and WS methods. Therefore, the SSF method was used to produce fermented rice milk for use as paneer coagulant.

### 3.3. Analysis of Paneer

#### 3.3.1. Ash Content and Total Yield of Paneer

Ash content determines the amount of total minerals in a food matrix. The ash content was measured only in fresh samples to examine the effect of rice milk addition to dairy milk. The total ash content was lower in control samples compared to the sample containing rice milk. This may be due to the higher carbon source ingredients contained in rice milk, as presented in Table 1. The ash content in this study was similar to the reported findings [3], where the ash contents was about 1.75% of paneer from indigenous coagulated milk.

The yield of paneer samples was measured in fresh samples after the whey was drained by muslin cloth. In optimum processing conditions, the yield of paneer was in the range of 19.44% to 21.67%; the results are presented in Table 1. The yield of the paneer decreased with the increase of the rice milk addition. The lowest yield was 19.44% in the 30% rice milk paneer samples, whereas the control sample showed a significantly higher yield (21.67%). This is due to the coagulation of protein and entrapped of SNF (solid not fat) and fat content of milk casein in control paneer [1]. In rice milk-containing samples, rice milk being additionally included with the dairy milk as a coagulant while rice milk protein cannot be coagulated as casein. Therefore, some portion of rice milk was drained with whey which lowered the yield of rice milk containing samples.

#### 3.3.2. Changes of Moisture during Storage of Paneer

The moisture content in a product is essential because higher water content allows the growth of micro-organisms, decreasing the shelf life of the product. The moisture content in paneer is usually high due to heat and acid expansion of casein micelles, which holds moisture [1]. Normally, paneer has 40% to 55% moisture content, affecting and limiting its shelf life to one day at room temperature [1]. The initial moisture content in this study from 0–30% rice milk containing samples were (40–41%), which show a lower moisture content than previously reported, which was (52.19%) [29]. The changes in moisture content during storage are presented in Table 2. The moisture content was 41.5% in fresh control samples, which reduced to 39.9% after 12 days of storage at 4 °C. Samples from 10% of rice milk showed the same trend of moisture content during storage. In 20% and 30% rice milk containing paneer samples, initial moisture content was 41.13% and 41.29%, and it reduced to 37.79% in both samples after 12 days storage due to higher bacterial count in 20% and 30% rice milk paneer samples compared to control. Another reason is the expulsion of moisture content from the product to its surroundings, which were greater in paneer with high percentages of rice milk used. This is due to the low-fat content, and the non-compact and fragile structure [3].

#### 3.3.3. Changes of Total Acidity during Storage of Paneer

The acidity of paneer increases during the storage period due to microbial activity, fat oxidation, and moisture loss, which influences the shelf life of products. To evaluate the storage life of paneer, the total acidity as LA equivalent was determined during storage, and the results are presented in Table 2. Total acidity of the control samples increased from 0.161 to 0.431 during storage of 12 days, whereas samples containing higher rice milk (30%) showed a significantly greater acidity range (0.371–0.657) during the same length of storage. Total acidity increased in initial samples with the amount of rice milk from 10% to 30%, due to higher a portion of rice milk containing higher LA. Previous studies have reported the increase of acidity during storage due to higher bacterial activity and fat oxidation [3,30].

#### 3.3.4. Microbial Growth during Storage of Paneer

Paneer shelf life is mainly affected by microbial growth on the surface of paneer during processing and storage. The microbial count was determined in a 12 day long storage period and results are presented in Table 2. The microbial count was significantly higher at initial storage period in a sample with 30% rice milk (4.9 log CFU g^−1^) compared to control samples (3.9 log CFU g^−1^). The microbial count of control samples was almost stable during storage. The results are comparable with the study of M.K. Agnihotri et al. [31], where the acceptable range for microbial count on paneer was reported as 4.47 to 5.39 log CFU mL^−1^. Contrarily, in samples with 20% and 30% of rice milk, the microbial count reduced in the first six days of storage and then increased throughout the storage duration. The high microbial count in rice milk paneer samples is due to the LAB present in rice milk, since fermented rice milk was only partially pasteurized (heated up to 70 °C) before addition to dairy milk. There were not any noticeable defects observed in stored samples at 4 °C for 12 days, and our findings agreed with the results reported [3], where the authors studied the shelf life extension of paneer by adding plant essential oil and using different packaging materials.

#### 3.3.5. Texture Analysis during Storage of Paneer

The texture is regarded as the manifestation of the rheological properties of food. The textural properties of paneer are one of the main elements which affect its favor. The changes in texture of paneer during storage are mainly due to microbial activities and loss of moisture content, leading to its either soft or hard body and texture [29]. Texture changes in paneer are due to the changes in milk protein during the heating and acid process [1]. This accounts for the firm structure and high moisture content that creates its soft body and texture of paneer. We analyzed the properties of fresh and stored paneer samples by its hardness. Table 2 shows the results of the hardness of fresh and stored paneer samples. The initial hardness of samples incorporated with rice milk was higher than those of the control due to the fibrous structure of rice milk and lower fat content, as rice milk contain a lesser amount of fat compared to dairy milk. The hardness of 20% and 30% rice milk samples increased after three days of storage and then decreased continually until the end of the storage. The increase of hardness of the rice milk paneer samples was due to the presence of resistance starch, increasing paneer hardness under refrigerator temperatures [32]. The results were comparable with Jain et al. [23] who analyzed the textural properties of soya paneer.

#### 3.3.6. Color Measurements during Storage of Paneer

The measurement of color shows food quality and the defect level of certain food. Usually, color measures with colorimeter L, a and b values can only detect a very small area of food. Recently, computer vision was used to measure food color which analyzes the entire surface of food and quantifies the defect surface amount. In the RGB scale measurement of color, the scanner detects the intensity of light in red (R), green (G), and blue (B) [33]. In this study, the RGB scale of color measurement was used to detect the color changes in fresh and stored samples of paneer. The values were then converted to CIE color parameter as L* a* and b* values, which are known to show similar responses to human vision [34].

Table 3 shows the results of color changes during storage, the fresh paneer exhibited light grayish white color. After storage (12 days) the color changed to a light grayish yellow, which was apparently observed as white. No significant color changes were observed for control samples after 12 days of storage. The color of paneer samples containing 10% of rice milk was not significantly different from control samples. Furthermore, the fresh sample from 20% rice milk was light grayish yellow and then further changed to grayish yellow during storage for 12 days. The color of 30% rice milk samples were lighter yellowish in fresh sample and the value reduced to a slightly grayish yellow during storage. Samples from 10% of rice milk were closer to control samples in color than 20% and 30%, due to the color of rice milk. The color values slightly decreased in all the samples during storage. These results were comparable with those previously reported [35] where the color properties were analyzed of soy paneer prepared from mixtures of cow skim milk and soymilk.

#### 3.3.7. Sensory Evaluation

**Figure 4:**
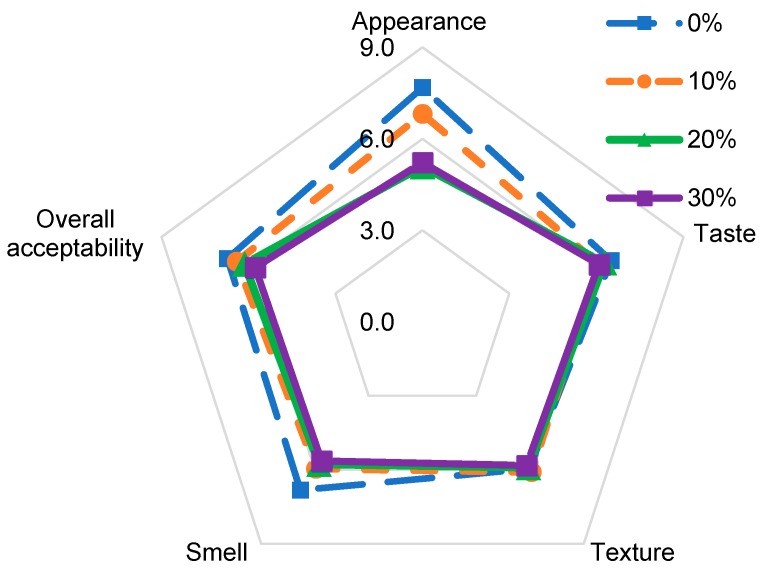
Sensory attributes of paneer control and rice milk incorporated samples.

Sensory evaluation was conducted to identify the acceptability of the fresh paneer samples. Figure 4 shows the results of sensory evaluation. The score for appearance, taste, texture, smell, and overall acceptability was higher in control samples. The score decreased for appearance from 7.7 in control to 5.2 in 30% samples. However, there was no significant difference between control and 10% rice milk paneer samples. Contrarily, there were no significant differences among all the samples in taste and texture. The samples were significantly different in smell from control and 10% in comparison to 20% and 30% due to the use of fermented rice milk. The score for overall acceptability reduced slightly, but there were no significant differences among all the samples, and on overall acceptability the product was rated overall acceptable by the panelists.

## 4. Conclusions

The fermentation of rice milk with the SSF method and its utilization as a novel coagulant for paneer production was demonstrated successfully. This study revealed that there were no significant differences among fermented samples, thus the SSF method was more efficient for rice milk fermentation due to less energy and time consumption. Paneer analysis and sensory evaluation indicated that fermented rice milk has a positive effect on the texture and shelf life of paneer. Paneer samples with a lower ratio of rice milk showed more or less similar properties to control samples, maintaining its original color and taste. Sensory evaluation revealed that paneer samples were acceptable for all the portions of rice milk and there were no significant differences when compared to the control in overall acceptability. This study succeeded in determining the optimal ratio of fermented rice milk and dairy milk for paneer making, and further work is needed to adjust the conditions to preserve its original properties as paneer for a commercial product.

## Figures and Tables

**Table 1:** Percentage of Ash and total yield of fresh paneer.

Rice Milk (%)	Ash Content (%)	Total Yield (%)
0	1.19 ± 0.016 ^c^	21.67 ± 0.24 ^a^
10	1.36 ± 0.009 ^c^	20.02 ± 0.31 ^b^
20	1.49 ± 0.002 ^b^	19.58 ± 0.25 ^b^
30	1.78 ± 0.005 ^a^	19.44 ± 0.99 ^b^

Each value of the Ash content and total yield is shown as the mean ± SD. The means with different lowercase superscripts in a column within each concentration differs significantly by Tukey test α = 0.05.

**Table 2:** Paneer properties changes during storage (±4 °C).

Storage Properties	Rice Milk (%)	Storage Period (Days)				
		1st	3rd	6th	9th	12th
Moisture Content (%)	0	41.51 ± 1.39 ^a^	40.35 ± 1.52 ^a^	40.13 ± 1.39 ^a^	40.04 ± 0.02 ^a,b^	39.93 ± 0.11 ^a^
	10	40.39 ± 0.69 ^a^	40.27 ± 0.28 ^a^	40.27 ± 0.02 ^a^	40.07 ± 2.51 ^a^	39.88 ± 0.23 ^a^
	20	41.13 ± 0.61 ^a^	40.53 ± 0.86 ^a^	39.47 ± 0.98 ^a^	39.25 ± 1.28 ^b^	37.79 ± 1.87 ^a^
	30	41.29 ± 0.74 ^a^	40.79 ± 1.99 ^a^	39.95 ± 0.55 ^a^	39.91 ± 0.51 ^a,b^	37.79 ± 2.27 ^a^
Total Acidity (%)	0	0.161 ± 0.007 ^a^	0.168 ± 0.007 ^a^	0.265 ± 0.040 ^a^	0.342 ± 0.050 ^a^	0.431 ± 0.070 ^a^
	10	0.175 ± 0.007 ^a^	0.155 ± 0.006 ^b^	0.157 ± 0.007 ^a,b^	0.165 ± 0.007 ^b^	0.158 ± 0.020 ^b^
	20	0.331 ± 0.004 ^c^	0.341 ± 0.008 ^c^	0.356 ± 0.006 ^b^	0.366 ± 0.017 ^b^	0.376 ± 0.006 ^b^
	30	0.371 ± 0.007 ^c^	0.378 ± 0.006 ^c^	0.456 ± 0.009 ^c^	0.557 ± 0.007 ^c^	0.657 ± 0.004 ^c^
Microbial growth (log CFU g^−1^)	0	3.9 ± 0.301 ^b^	3.7 ± 0.113 ^b^	3.8 ± 0.030 ^a^	3.8 ± 0.069 ^a^	4.1 ± 0.003 ^a^
	10	4.1 ± 0.088 ^b^	3.9 ± 0.053 ^ab^	3.8 ± 0.668 ^a^	4.1 ± 0.763 ^a^	4.1 ± 1.398 ^a^
	20	4.3 ± 0.140 ^a,b^	3.9 ± 0.326 ^a,b^	3.7 ± 0.899 ^a^	4.1 ± 0.476 ^a^	4.5 ± 0.498 ^a^
	30	4.9 ± 0.041 ^a^	4.4 ± 0.316 ^a^	3.7 ± 0.659 ^a^	4.5 ± 0.667 ^a^	4.6 ± 1.220 ^a^
Hardness (N)	0	4.56 ± 0.03 ^a^	4.11 ± 1.05 ^a^	6.78 ± 0.06 ^a^	6.23 ± 0.08 ^a^	5.36 ± 0.53 ^a^
	10	6.02 ± 0.44 ^a^	11.30 ± 0.25 ^a,b^	7.24 ± 0.13 ^a^	10.54 ± 1.11 ^a,b^	8.04 ± 0.58 ^a^
	20	4.48 ± 0.43 ^a^	8.16 ± 0.07 ^b^	7.04 ± 1.18 ^a^	8.07 ± 1.05 ^ab^	7.68 ± 0.18 ^a^
	30	5.43 ± 0.66 ^a^	9.38 ± 0.26 ^c^	7.04 ± 0.47 ^a^	8.57 ± 1.00 ^b^	7.49 ± 0.34 ^b^

Each value shown as the mean ± SD. The means with different lowercase superscripts in a column within each concentration differs significantly by Tukey test α = 0.05.

**Table 3:** Changes of color of paneer during the storage period.

Rice Milk (%)	Storage Period (Days)					
	Color Parameter	1st	3rd	6th	9th	12th
0	L*	97.910 ^a^	98.514 ^a^	97.076 ^a^	98.912 ^a^	90.675 ^a^
	a*	−0.369 ^a^	−0.984 ^a^	−1.252 ^a^	−1.186 ^a^	−1.981 ^a^
	b*	1.373 ^a^	1.412 ^a^	−0.128	3.068 ^a^	1.193 ^a^
10	L*	98.222 ^a^	97.117 ^a^	93.812 ^a,b^	96.369 ^a^	92.075 ^b^
	a*	−1.022 ^a^	−0.945 ^a^	−1.809 ^a^	−1.238 ^a,b^	−0.542 ^a^
	b*	4.284 ^a,b^	3.059 ^a^	0.725 ^a,b^	4.762 ^a,b^	2.907 ^a^
20	L*	95.712 ^a^	96.470 ^a^	97.023 ^a,b^	95.988 ^a^	91.404 ^a,b^
	a*	−1.227 ^a^	−1.218 ^a,b^	−1.281 ^a^	−1.354 ^a,b^	−1.348 ^a^
	b*	7.342 ^b^	6.653 ^b^	7.337 ^b,c^	7.817 ^b,c^	2.707 ^b^
30	L*	94.380 ^a^	94.072 ^a^	93.371 ^b^	95.848 ^a^	91.702 ^a^
	a*	−1.785 ^a^	−1.394 ^b^	−2.060 ^a^	−1.643 ^b^	−0.134 ^a^
	b*	13.858 ^c^	9.395 ^c^	9.457 ^c^	11.455 ^c^	7.392 ^a^

Each value of the color is shown as the mean ± SD. Colors with different lowercase superscripts in the same column are significantly different (α *=* 0.05) as the letters compare the data with similar color values, for instance, L* compared with L*, a* with a*, and b* with b*.
